# Narrow-Band Imaging in Digestive Endoscopy

**DOI:** 10.1100/tsw.2007.99

**Published:** 2007-03-30

**Authors:** R. Lambert, K. Kuznetsov, J-F. Rey

**Affiliations:** ^1^ Institut Arnault Tzanck, St Laurent du Var, France; ^2^ International Agency for Research on Cancer, Lyon, France

**Keywords:** endoscopy, esophageal cancer, stomach cancer, colon cancer, esophagogastric junction, celiac disease

## Abstract

The application of opto-electronics in video-endoscopes improves the accuracy in diagnosis, through image processing and digital technology. Narrow Band Imaging (NBI), consists of using interference filters for the illumination of the target in narrowed blue and green bands of the spectrum. NBI is combined with magnifying endoscopy using an objective macro or an optical zoom. The NBI technique developed by Olympus Medical Systems is now available in the most recent models of video-endoscopes that use the non-sequential system of illumination (Lucera Spectrum) or the sequential R/G/B system of illumination (Exera II). The major contribution of the technique is in the characterization (analysis after detection) of the flat and superficial neoplastic areas of the digestive mucosa, with a specific application to the identification of intestinal metaplasia and early neoplastic changes in the Barrett's esophagus. The technique also proves helpful for the assessment of the vascular pattern in chronic inflammatory disorders of the digestive mucosa.

## INTRODUCTION

In developed countries, Health authorities are now concerned with the prevention of cancer, because a significant increase of the burden is expected to occur during the next two decades in the aging population of the world. Therefore the incidence and mortality are expected to increase for cancer in the esophagus, stomach and large bowel. While primary prevention addresses to a healthy style of life and the control of causal factors, the secundary prevention of cancer is based upon early detection at an early and curable stage. In the digestive mucosa the precursors of advanced cancer include premalignant benign lesions and malignant lesions (superficial cancer). The endoscopic appearance of superficial neoplasia (intramucosal or submucosal) in the digestive mucosa has been codified in subtypes of a type 0 in the Paris classification derived from the Japanese classification[1]. Polypoid (0-I) lesions are conspicuous and easily detected, even when small. Non polypoid (0-II) lesions, slightly elevated (IIa), completely flat (IIb) or slightly depressed (IIc), are less conspicuous, requiring adaptation of our cognitive appreciation of what is abnormal in the visual image, i.e. a simple discoloration or a variation in the network of subepithelial capillaries[2]. Those lesions are easily missed at endoscopy and are frequent: it is estimated that they account for 80% of superficial neoplasia in the esophagus, 95% in the stomach and 45% in the colon[1].

Recently a considerable technological advance occurred in both sectors of optics and electronics of the video-endoscopes, reaching the status of high-resolution endoscopy. The optical zoom offers an access to the fine structure of the mucosa including the surface of the epithelium and the superficial vascular network. The digital reconstitution of the image captured by the video-endoscope, opens the way for image processing with structure enhancement through modulation in the amplitude of specific wavelengths in the reflected light and with Index of haemoglobin (IHb) through modulation of the color of the reflected light. The narrow-band imaging (NBI) techniqus, coupling magnifying endoscopy and image processing, introduces a new era in diagnostic endoscopy. In the instrument developed by Olympus Medical Systems, the incident light is distributed in discontinuous narrow bands of photons, which have distinct depths of penetration in the mucosa and submucosa. A selective enhancement of the epithelial crests and depressions at the surface of the mucosa is obtained, as well as a contrasted image of the subepithelial capillaries. The major contribution of the NBI technique concerns the detection and characterization of the nonpolypoid superficial neoplastic lesions in the digestive mucosa. Imaging of chronic inflammatory lesions and their capillary network is also improved.

## THE NARROW-BAND IMAGING TECHNIQUE

### Image Reconstitution in Video-Endoscopes

Video-endoscopes use the white light of the Xenon source for illumination. The depth of penetration of the incident photons in the digestive wall depends on their wavelength: superficial for the blue band, in depth for the red band and intermediate for the green band. Hemoglobin is the major responsible agent for the absorption of the blue and green components of the visible light; this explains the red color of the vessels in the reflected light captured by the CCD chip at the tip of the instrument for image reconstitution. The CCD is coupled to a processor and a single natural color image is reconstructed from the three red, blue and green (R/G/B) monochromatic images and displayed on the color monitor. Alternatively the color image is reconstructed from the colors cyan, magenta, yellow and black (C/M/Y/K). The spectral composition of the reflected light is influenced by the laminar structure of the digestive mucosa, which acts as a scattering element and interferes with the reflectance spectrum.

Two different systems are used in the image reconstitution by the processor. In a "color" CCD, pixels are selectively attributed to specific wavelength ranges, such as red, green and blue (R/G/B). The CCD transfers, in a single step (non-sequential system of the EXERA series, Olympus Medical Systems Corp.), the full range of the white light to the processor for reconstitution of the natural color in the video-monitor. In a “monochrome” CCD, pixels are not selectively attributed to specific colors and the mucosa is illuminated alternatively in each of the 3 bands because a rotating interference broadband (R/G/B) filter is interposed after the white light of the Xenon lamp. Its rotation maintains the full range of the visible light between 390 and 655 nm. The reflected photons are transferred successively in the R/G/B bands to the processor (sequential system of the LUCERA series, Olympus Medical Systems).

### Principles of the NBI technique

In the Narrow Band Imaging technique (NBI, Olympus Medical Systems Corp.) a special set of filters is interposed after the light source to restrict the incident light in two narrow bands (figure 1) of wavelengths (blue at 415 nm and green at 540 nm). The same bands are used in the 2 available NBI systems (EXERA II and LUCERA SPECTRUM), which only differ, by the scanning mode of the reflected image (sequential versus simultaneous). The selective reflection of the NBI light by the superficial layers of the mucosa improves the definition of the surface; while its selective absorption by hemoglobin, enhances the contrast of the vascular network (figure 2). Therefore the most specific contribution of NBI is the enhancement of the microvascular network of the normal and neoplastic

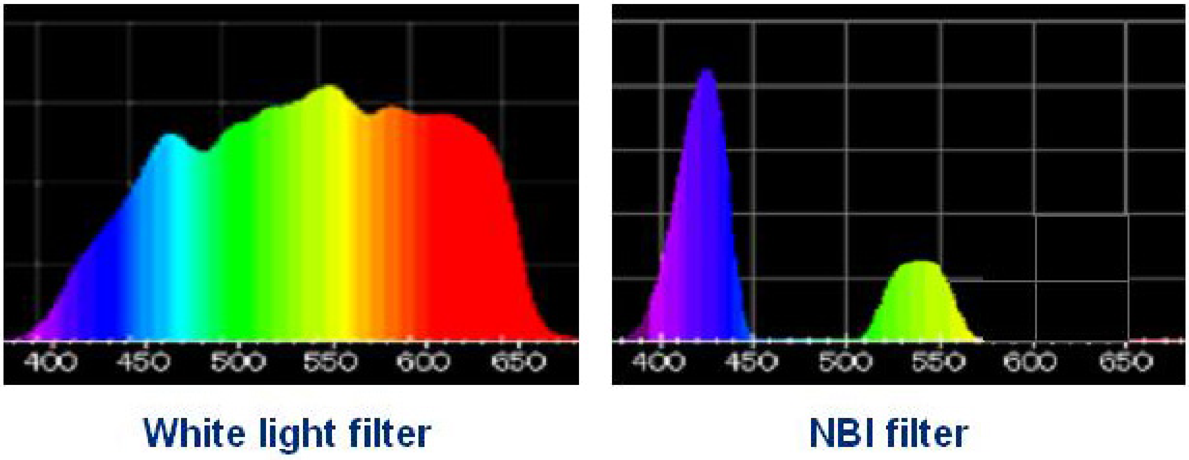

**FIGURE 1.** Reproduction of the radiance of the spectrum obtained when using the broadband (white light) interference filter in the range 400 to 650 nm, or the narrowband (NBI light) interference filter, with 2 distinct peaks, blue at 415 nm and green at 540 nm. Note the higher radiance of the blue band. *(courtesy of Olympus Corporation, Japan).*

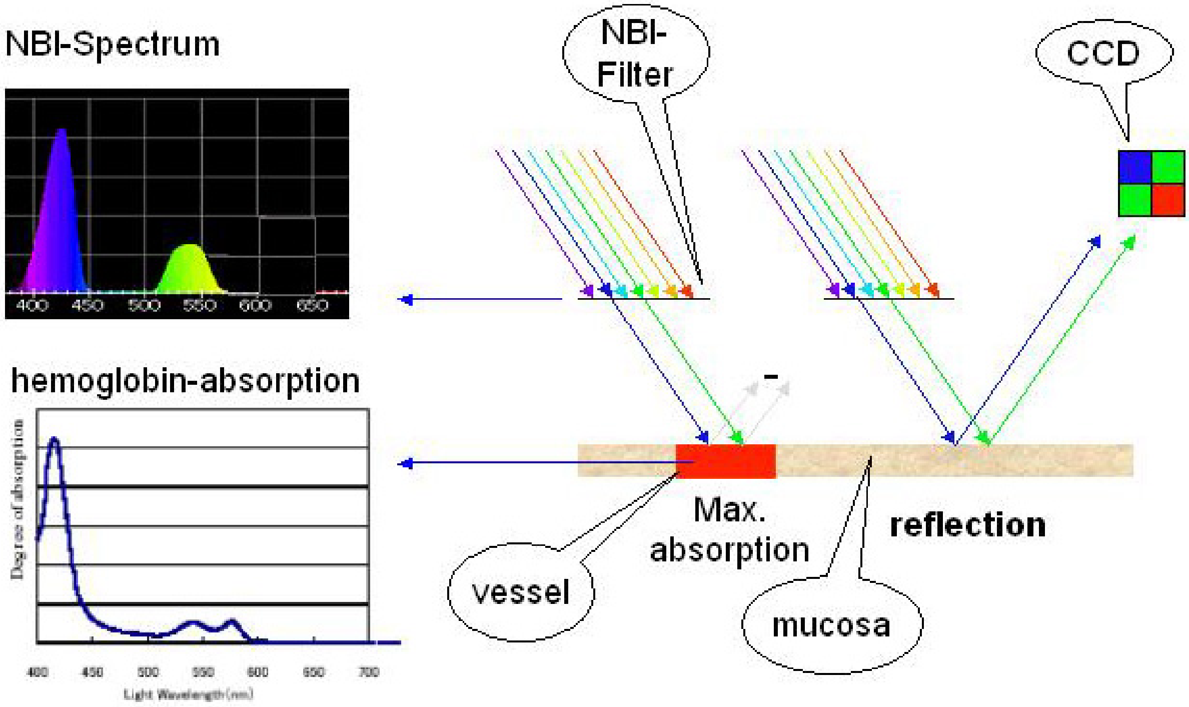

**FIGURE 2.** Schematic representation of the interactions between tissue and light when using the NBI interference filter: the blue and green bands are absorbed by hemoglobin in the vessels and reflected by the surface of the mucosa. The peak of absorption of hemoglobin is in the blue *(courtesy of Olympus Corporation, Japan).*

mucosa. The two reflected images from the surface of the mucosa, (415 nm) and from the depth of the mucosa and the submucosa (540 nm) are collected. The 415 nm image channel analyzes the fine surface architecture of the mucosa and the superficial capillary network; the 540 nm image channel analyzes the collecting vessels in the depth of the mucosa. In the final mixed image, the processor further enhances the contrast by re-assigning the color channels: superficial and deep details are superposed in a single image, enhancing the visibility of flat lesions, while sub-epithelial capillaries are displayed in brown and veins in the submucosa in cyan. The video-monitor can alternatively display the standard colored image or the image in NBI.

## NBI Technique and Structure Enhancement

Two types of structure enhancement are available in the EXERA II, Olympus Medical Systems Corp.:

- the original type A designed for observation in high contrast of the epithelial structures (crests and depressions) is at its maximum all along the procedure[3]
- the new type B designed for observation of vascular structures may be activated at intervals during the examination.

## NBI Technique and Magnification

Magnification combines optical and electronic technologies to depict the fine structure (pits and ridges) of the surface of the epithelium in the esophagus, stomach and colon and the network of subepithelial capillaries.

The magnifying power of the optical zoom can reach up to x 150, but x 80 is enough for most indications; when the zoom is activated, the focal distance between the objective and the mucosal surface decreases in proportion to the power of magnification, therefore the tip of the instrument is placed at a very short distance of the target and a small area of the mucosa is explored. A transparent hood, fixed at the tip of the endoscope, helps to maintain an adequate and focused distance, particularly in the cardia region. The objective “macro”, less powerful is easier to use because the vision remains clear when the distance between the objective and the target varies; therefore no adjustment of the lens is needed.

With the electronic zoom the resolution loss does not affects practical examination, if the image is processed in the HDTV standard.

NBI is coupled to magnification, either with an objective “macro” (EXERA II, Olympus Medical Systems Corp.), and the electronic zoom, or with an optical zoom (LUCERA SPECTRUM, Olympus Medical Systems Corp.).

## NBI Technique and Chromoscopy

When the surface of the mucosa is explored in NBI, spraying a colored contrast agent is not recommended. This applies particularly to the analysis of subepithelial capillaries where the procedure is always conducted in “transparency”, without dye spraying.

The NBI technique is often considered as a substitute to chromoscopy because the color of the mucosa in changed and the contrast between a neoplastic area with the surrounding mucosa is enhanced[3]. However there is stillroom for dye spraying on the surface of the mucosa. Chromoscopy with indigocarmine is still the simplest procedure to describe slight elevations and depressions of the surface for the classification of the morphology of the neoplastic lesions in type 0. Chromoscopy with indigocarmine or cresyl violet is also adapted to the description of the pit pattern with the optical zoom. Spraying of acetic acid (3% solution) a non-colored contrast agent may prove helpful in the analysis of the esophago-gastric junction[4]; however it should be avoided for the analysis of the vascular network because it provokes a vascular congestion impairing the precise analysis of the capillary network.

Artificial chromoscopy has recently proposed by Fujinon using the computer reconstitution of virtual and sharp images at specific wavelengths (FICE, EPX 4400). The technique and results differ from NBI in the following the reconstituted images are extracted from the reflected photons issued from incident photons, which display the full broadband of the white light.

## ENDOSCOPIC DIAGNOSIS WITH NARROW-BAND IMAGING

### Conducting the Endoscopic Procedure

There is no special requirement for the patient's preparation and sedation. The first step, conducted in standard observation, aims to identify landmarks and any abnormality of the surface. Then the specific points of interest are explored with the objective macro or the optical zoom. The magnified image is successively switched from normal color to NBI function. This means that the NBI function is not maintained all along the procedure, but used at intervals on selected targets.

As compared to the standard image (in white light) displayed on the monitor, the image in NBI is less bright and colored in brownish grey; on the other hand, the contrast of the surface and that of the vascular network is far superior. Still-magnified pictures of zones with specific characters are automatically captured on the computer hard disc, in standard and NBI colors, when pressing the endoscope freeze button. The reliability of the findings relies on the comparison of the recorded images with histology and the patient's history.

### Strategy for Endoscopic Diagnosis

High-resolution endoscopy with NBI is not a substitute to the careful exploration of the mucosa; the variations in the appearance of the mucosa require an extreme attention in order to discriminate between, non clinically relevant, and relevant artifacts. There are two steps in endoscopic diagnosis: detection and characterization.

The step of detection is conducted without image processing in standard vision. Polypoid neoplastic lesions have a conspicuous appearance, but nonpolypoid lesions are easily missed if the operator is not aware of a slight change in the color of the mucosa or an interruption of the subepithelial vascular network at the line of demarcation.

Characterization after the step of detection avoids unnecessary resection of lesions with a very low malignant potential, or inappropriate endoscopic treatment of lesions, which should be treated directly by surgery. Characterization relies on the following elements:

1 - the configuration of the lesion: this step is extremely important and does not require magnification. The limits and relief (elevation and depression) are assessed, still in standard vision, with the help of chromoscopy and superficial neoplastic lesions are classified in subtypes of type 0-I, 0-II or 0-III.

2- the microvascular network around and at the surface of the lesion: this is best explored in "transparency" (no chromoscopy) with enhanced contrast and with NBI coupled to magnification at a low power. The major contribution of the NBI technique is in the analysis of the subepithelial vessels, which tends to be predictive of histology; the alterations of the pattern of subepithelial capillaries can be classified as slight or severe. Slight alterations suggest a non-neoplastic lesion or low-grade dysplasia. Severe alterations suggest high-grade dysplasia or cancer.

3 - the fine structure of the epithelium in surface is best explored in magnification with an optical zoom at a high power (x 80 or x 100), either in NBI or without image processing and the help of indigocarmine chromoscopy. In a columnar type of epithelium when severe vascular alterations are detected at a low magnification, the "pit pattern" of the surface should be explored at a higher magnification power, either using chromoscopy or the NBI technique.

## NBI IN THE ESOPHAGUS

In the esophagus the surface of the normal stratified squamous epithelium has a smooth appearance with a clear pink color. In magnification multiple red dots correspond to the intrapapillary capillary loops (IPCL), which have a regular pin-hair shape.

The easiest procedure to detect flat neoplastic areas in the squamous epithelium and evaluate their surface is still the spraying of an iodine-potassium-iodide solution (Lugol). The morphology of the lesion classified in type 0 has some predictive value for the depth of extension (intraepithelial neoplasia versus invasive cancer). However the pattern of subepithelial capillaries (IPCL) explored in magnification in transparency without chromoscopy is more reliable for prediction: 5 distinct patterns of vessels have been described[5] and a study in operative specimens has confirmed a positive relation between the increasing diameter of superficial vessels and histological progression to neoplasia. Actually a description of the microvascular network in 3 categories proves relevant for clinical practice: 1 - when the pin-hair pattern of the IPCL is maintained, while they are dilated and elongated; this suggests esophagitis or low-grade intraepithelial neoplasia 2- when the pin-hair pattern disappears and is replaced by a punctuated pattern where the capillaries agglomerate in dense packages and show tortuosity, irregularities in the diameter; this suggests high grade intraepithelial neoplasia or carcinoma *in situ*. 3- when the punctuated pattern is replaced by multiple large and irregular neo-vessels; this suggests invasive intramucosal or submucosal carcinoma.

NBI coupled to magnification introduces a definite progress in the characterization of superficial neoplastic lesions in the stratified squamous epithelium. The color contrast-ratio between vessels and mucosa is greater for the NBI image: the capillary loops are more visible, with a dark color; in addition large drainage vessels, colored in cyan are visible in the subjacent *lamina propria* (figure 3). There is a trend to develop T staging of superficial cancer through the endoscopic evaluation of the depth of invasion. In a study conducted in 41 patients[6], the endoscopic evaluation of the depth of invasion in 3 layers for mucosa (m1, m2, m3) and in 3 layers for submucosa (sm1, sm2, sm3) on NBI images , was compared to the pathology controls and the overall accuracy reached 85.2 % for experienced endoscopists.

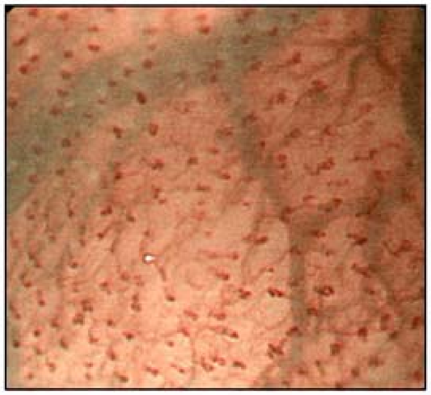

**IPCL + collecting veins**

**FIGURE 3 -** Esophago-gastric junction; squamous epithelium in the distal esophagus; narrow-band imaging coupled to Magnification. The dark brown dots correspond to the top of the intrapapillary capillary loops. The larger vessel colored in cyan is a collecting vein more in depth.

## NBI AT THE EG JUNCTION AND IN BARRETT ESOPHAGUS

The esophagus joins the stomach at the level of the diaphragmatic pinch, in the absence of hiatal herrnia. Esophagus is lined by a stratified squamous epithelium and stomach by a columnar epithelium. In standard endoscopic vision, the exploration of the esophago-gastric (EG) region is based on landmarks such as the hiatal pinch, gastric folds, palisade vessels and is completed by the analysis of the proximal and distal verge of the Z line. Useful complements in the analysis of the Z line include spraying of an iodine-potassium-iodide solution (Lugol) or an acetic acid solution (figure 4). The squamo-columnar epithelial junction, or Z line, is located just above the pinch of the diaphragm. The EG region includes the distal part of the esophagus and the proximal part of the stomach or cardia. Just above the Z line characteristic features of the stratified squamous epithelium have been described by the Japanese endoscopists and pathologists: the palisade vessels and small islands of ectopic cardiac mucosa, resulting from the overlap of squamous epithelium over columnar epithelium. Just below the Z line the upper pole of the longitudinal folds of the gastric mucosa marks the proximal limit of the stomach, with a short (2 to 6 mm) segment of gastric cardiac mucosa in transition to the oxyntic (fundic) epithelium (figure 5). When there is a proximal shift of the Z line in the thorax, a segment of esophagus is lined with metaplastic columnar epithelium, also called Barrett esophagus, which comprises distinct epithelial types: gastric cardiac, oxyntic and incomplete intestinal metaplasia. When the endoscopic landmarks are approximately in place, an ultrashort (less than 1 cm) segment of metaplasia, above the gastric cardiac mucosa, can be suspected if palisade vessels and/or small islands of squamous epithelium are visible just below the Z line. Indeed these are anatomical features characteristic of the esophagus.

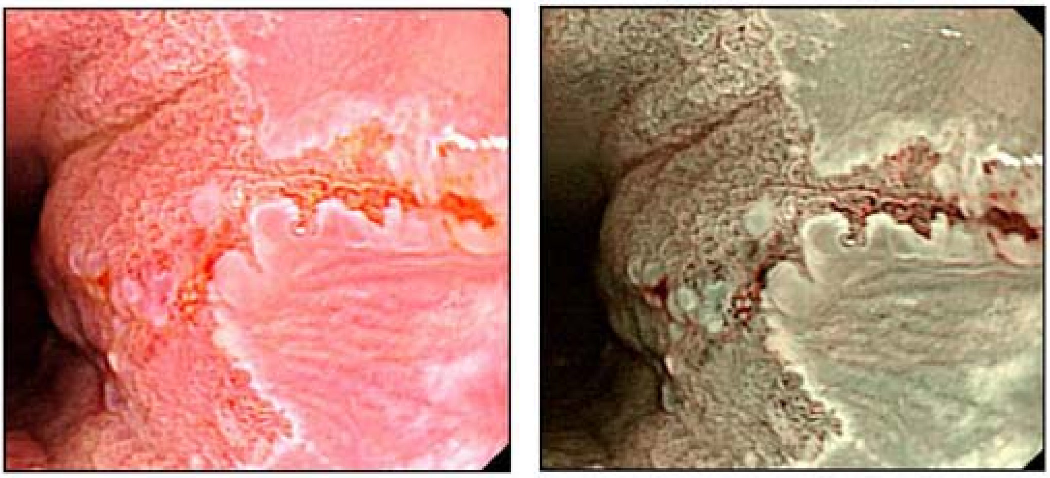

**FIGURE 4.** (a) Esophago-gastric junction, standard image, no magnification, after spraying of the acetic acid solution. The contrasted Z line is interupted by a short digitation of gastric cardiac epithelium in the distal esophagus, with a visible linear longitudinal erosion; (b) Esophago-gastric junction, same image as in Figure 3a in narrow-band imaging. The fine structure of the gastric cardiac epithelium is more contrasted.

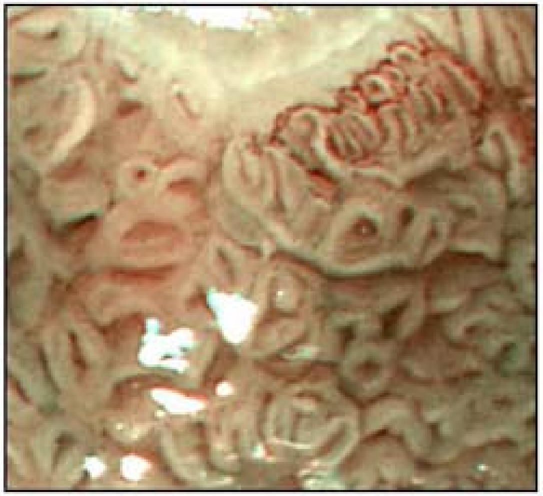

**Cardiac mucosa NBI**

**FIGURE 5.** Esophago-gastric junction; gastric cardiac epithelium under the Z line: narrow-band imaging coupled to magnification. Epithelial ridges and sulci highly contrasted.

During upper GI endoscopy good practice includes a systematic and careful exploration of the EG region in direct vision and in retrovision after a U turn of the tube in the stomach. The clinical relevance of this step links to the detection of Barrett esophagus and early detection of junctional adenocarcinomas which arise either from the esophagus or from the stomach; the burden of adenocarcinoma at the EG junction is the sum of cases at the gastric cardiac and in the lower third of esophagus. Early endoscopic detection is based on the identification of areas with intestinal metaplasia and adjacent flat areas with intraepithelial neoplasia. Standard endoscopy has a poor efficacy in achieving both objectives, even with the help of contrast agents (methylene blue, acetic acid). Therefore columnar epithelium in the esophagus or at the EG junction is the privileged target of endoscopy in magnification. The technique proved able to describe distinct patterns of epithelial crests and grooves for the fundic, cardiac, and intestinal types of columnar epithelium; irregularities of the fine structure were also shown in flat neoplastic areas. However the reliability and correspondence with histology is incomplete and if there is any doubt, multiple and blind tissue sampling in the segment with columnar metaplasia (Seattle protocol) is still recommended.

The exploration of Barrett esophagus and of the EG junction is one of the best indications of NBI coupled to magnification (figures 6-9).

1 - The analysis of the Z line and adjacent areas is improved with striking contrast between the stratified squamous epithelium and the columnar epithelium, enhanced contrast of the palisade vessels and access to the fine structure of the gastric cardiac mucosa often altered by carditis.

2 - Mapping the mosaic of epithelial types in the Barrett esophagus is more precise in NBI and the detection of intestinal metaplasia is more reliable than when using only magnification. There is progress in the classification of the epithelial types with their histological correspondence: villous, cerebroid and ridged patterns have a high predictive value for intestinal metaplasia.

3 - In the columnar lined esophagus characters highly suggestive for the presence of a flat area with neoplasia include an abrupt change in the average size of the epithelial crests or their irregular and distorted pattern and areas with an amorphous surface. Variations of the microvascular network with abnormal vessels have been described in areas with dysplasia[9,12]; further studies are needed to decide whether their description and classification has relevance to the depth of invasion and therefore to the decision of endoscopic treatment. Now a day the question arises, but is still in debate, whether selective biopsies could substitute the Seattle protocol?

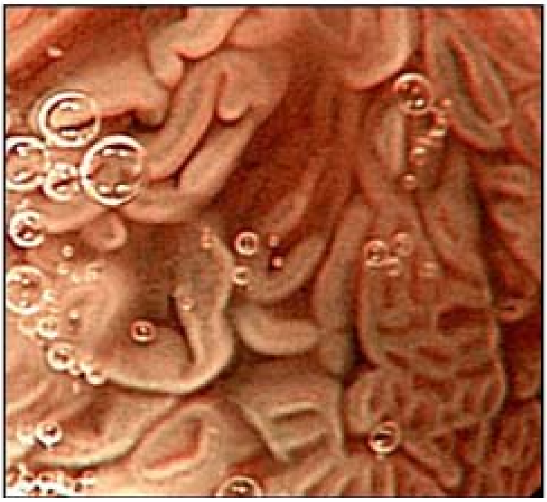

**Intestinal metaplasia in BE**

**FIGURE 6.** Esophagus, Barrett: Intestinal metaplasia; narrow-band imaging coupled to magnification. The epithelial crests show a ridged pattern

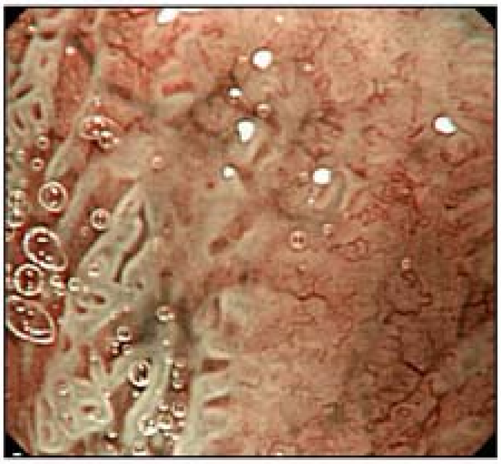

**LGD in BE, NBI**

**FIGURE 7.** Esophagus, Barrett: narrow-band imaging coupled to magnification. Left of the image; intestinal metaplasia with a ridged pattern. Right of the image. low-grade dysplasia in a zone with an amorphous appearance and an increased visibility of small dark brown vessels.

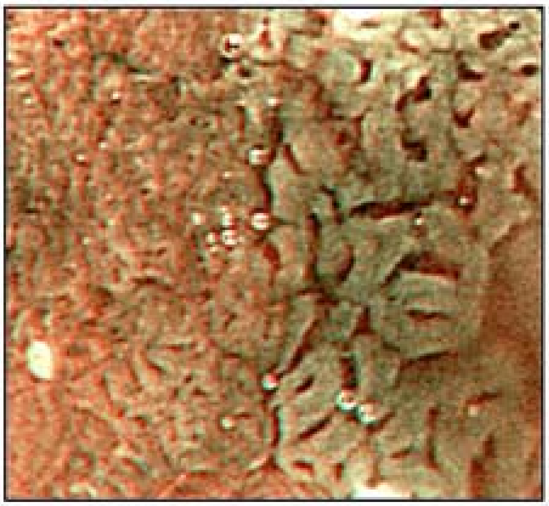

**HGD in BE, NBI**

**FIGURE 8.** Esophagus, Barrett: narrow-band imaging coupled to magnification. Right of the image; intestinal metaplasia and a ridged pattern. Left of the image high grade dysplasia in a zone with very small crests and pits

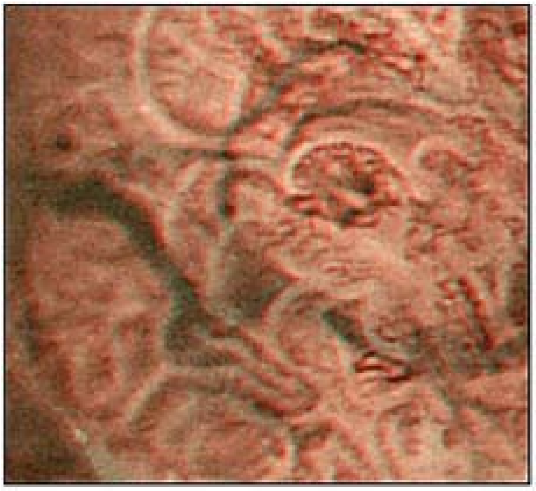

**Vessels in HGD, BE, NBI**

**FIGURE 9.** Esophagus, Barrett: narrow-band imaging coupled to magnification. High-grade dysplasia in a zone with irregular enlarged and branched epithelial crests. Vessels with a large and irregular caliber are colored in dark brown.

## NBI IN THE STOMACH

The fine structure of the gastric mucosa, in the absence of *H pylori* infection, has been amply described with the help of the optical zoom[16,17]. A further increase in precision is achieved when the NBI technique is coupled to magnification. The distinct patterns in the fundus (small pit openings) and in the antrum (ridges separated by narrow sulci) are more contrasted (figures 10-11). In the fundic mucosa, the subepithelial capillaries, surrounding the gastric pits, are organized in a regular honeycomb pattern, while in the antrum coiled vessels are at the centre of the epithelial ridges. More in depth the collecting veinules are disposed in a starfish design called the regular arrangement of collecting veinules (RAC). The subepithelial capillaries are colored in brown, and the collecting veins in cyan.

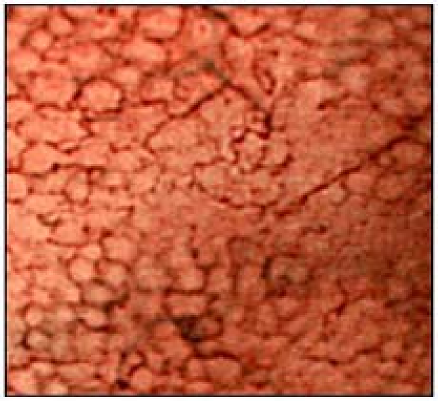

**Fundic mucosal and capillary network**

**FIGURE 10.** Stomach: Oxyntic mucosa in the fundus; narrow-band imaging at low magnification. Supepithelial capillaries with a honeycomb pattern around the opening of the gastric pits. Collecting veins colored in cyan with a starfish design.

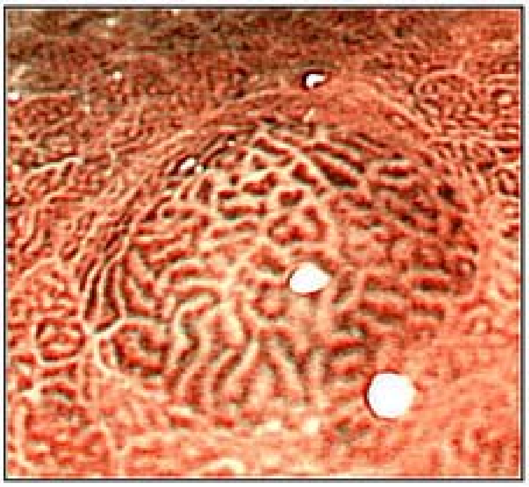

**Antral mucosa and island intestinal metaplasia**

**FIGURE 11.** Stomach: Antral mucosa in the distal stomach; narrow-band imaging at low magnification. A slightly protruding island of intestinal metaplasia with large and sinuous crests is surrounded by the shorter epitelial crests of the antral mucosa.

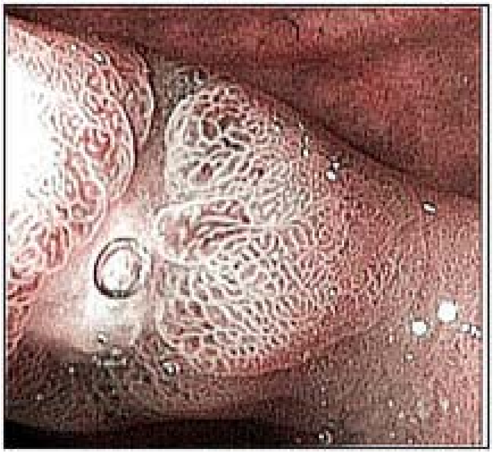

**Gastric ulcer, NBI**

**FIGURE 12.** Stomach: gastric benign ulcer in the antral mucosa; narrow-band imaging at low magnification. The architecture of the antral mucosa is well contrasted

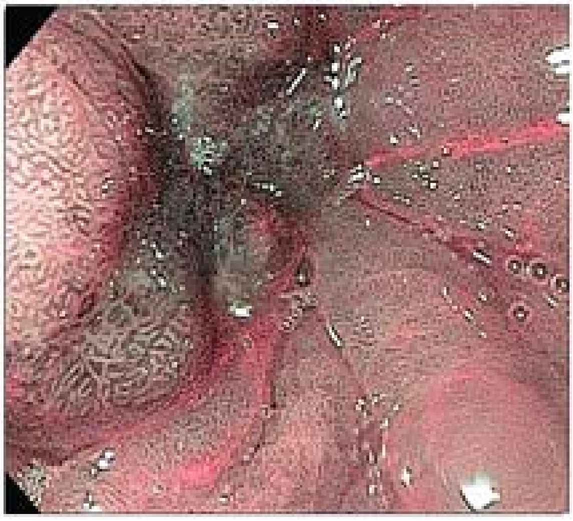

**Early gastic cancer in antrum**

**FIGURE 13.** Stomach: early gastric cancer in the antral mucosa; narrow-band imaging without magnification. Depressed lesion type 0-IIa with intestinal metaplasia and an irregular pattern of epithelial ridges.

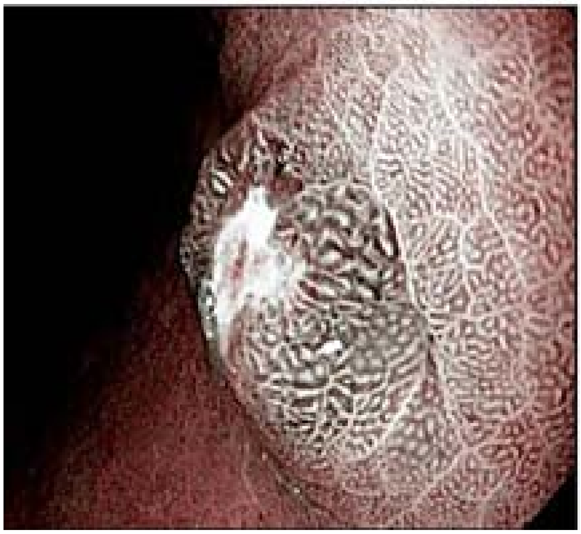

**stomach: Carcinoid NBI**

**FIGURE 14.** Stomach: gastric carcinoid with a central ulceration; narrow-band imaging coupled to magnification. In the antral mucosa the organization of the epithelial crests in large areas separated by deep sulci is enhanced.

## Gastric Neoplastic Lesions (figures 12-14)

Almost all precursors of advanced cancer have a non-protruding morphology and the depressed lesions (0-IIc) are from far the most frequent and can be misdiagnosed for benign gastric erosions or ulcer scars. In Japan since 40 years, taking in account the high burden of gastric cancer in this country, a priority has been attributed to the endoscopic description of those nonpolypoid lesions. The recent video-endoscopes allowed to describe the fine structure at the surface of the depressed neoplastic lesions in magnification coupled to indigocarmine chromoscopy. A irregular pattern of the epithelial crests (villous or gyrus) is described and zones with an amorphous pattern, are suggestive of invasive cancer. More recently the pattern of the vascular network in neoplastic areas has been described[18,19]. Intramucosal cancer shows superficial abnormal vessels (mesh, coil or corkscrew) depending on tumor differentiation. No doubt that the NBI technique coupled to magnification[18,19] will give a further impulsion to the description of the vascular pattern, aiming to predict invasion of the submucosa and its depth of invasion (superficial or deep), which is relevant to treatment decision.

## Gastritis

In *H pylori* infection the alteration of the surface architecture and of the vascular network of the gastric mucosa has been described in magnification. *H. pylori* infection causes a diffuse reddening of the surface of the oxyntic mucosa with regression of the honeycomb pattern and of collecting venules[15]. This applies to the endoscopic diagnosis of gastritis and to the regression of the abnormalities after eradication of *H pylori*. The analysis of the vascular network is expected to be more precise when magnification is coupled to NBI.

In confirmed chronic gastritis with atrophy, the surface fine structure of the mucosa is also altered by the occurrence of large zones with a depressed surface or an amorphous appearance. The major contribution of magnification coupled to NBI is then the detection of areas with intestinal metaplasia, where large and elongated epithelial crests separated by deep sulci, differ from the more superficial and shorter epithelial crests of the antral mucosa.

## NBI IN THE DUODENUM

The NBI technique, coupled to magnification proves helpful in patients with a gluten enteropathy (figures 15, 16), for the classification in degrees of atrophy of the duodenal villi, and for the evaluation of the response to treatment. When the atrophy is complete the vascular network is contrasted in a flat surface. Other applications concern the alterations of the mucosa in lymphoma and the characterization of ampullary tumors[20].

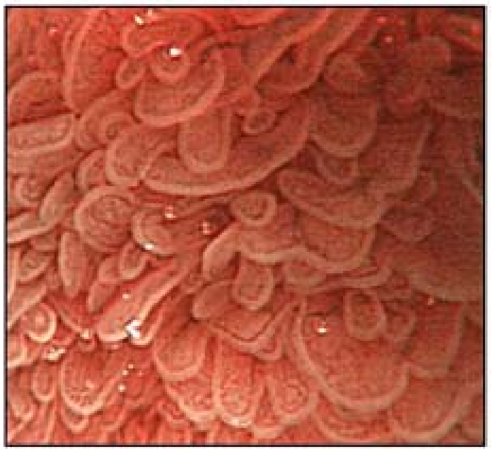

**Duodenal villi, NBI**

**FIGURE 15.** - Duodenum; villi of a normal size; narrow-band imaging coupled to magnification.The central axis of each villi is appearent.

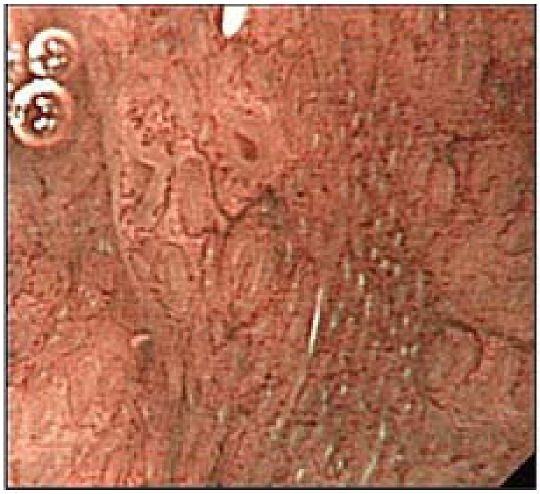

**gluten enteropathy, villous atrophy, NBI**

**FIGURE 16.** Duodenum: Gluten enteropathy with complete villous atrophy; narrow-band imaging coupled to magnification. The surface of the mucosa is smooth and the vascular network, colored in dark brown is visible

## NBI IN THE LARGE BOWEL

### Colorectal Neoplastic Lesions

Superficial neoplastic lesions in the large bowel are reliably detected using a high-resolution video-endoscope coupled to chromoscopy (indigocarmine) and their morphology is classified in the subtypes of type 0 lesions. The non-polypoid depressed lesions (0-IIc) frequently progress in the submucosa, even when their size is small. The pit pattern (figures 17, 18) analysed with the optical zoom has been classified in the 5 groups of Kudo distributed in 3 categories with clinical relevance: types I and II for normal or non-neoplastic - type III for low grade intraepithelial neoplasia - types IV and V for high grade intraepithelial neoplasia or cancer.

NBI coupled to magnification at a low power[21,22], proves extremely useful in routine endoscopy because of the easy distinction of the pit pattern in neoplastic and in non neoplastic lesions. In neoplastic depressed lesions when there is suspicion of high-grade intraepithelial neoplasia or invasive cancer, magnification at a high power coupled to indigocarmine chromoscopy can be used for a more precise analysis in order to select the therapeutic option.

The characterization of colorectal neoplastic lesions is without doubt the more frequent application of the NBI technique because the prevalence of those lesions in persons aged over 40 years is fairly high.

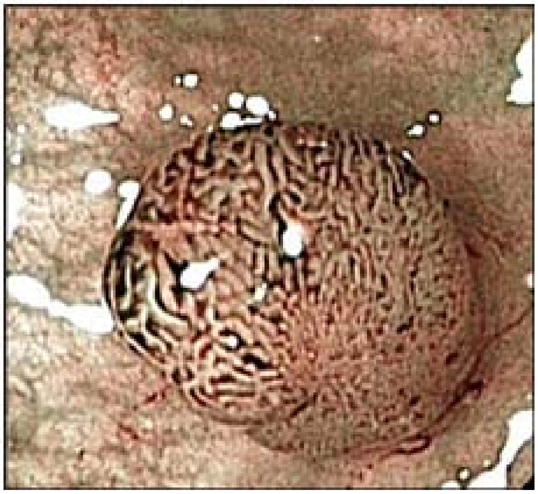

**Adenomatous polyp, type III L NBI**

**FIGURE 17.** Colon “ sessile small adenomatous polyp; narrow-band imaging coupled to magnification. Pit pattern classified as III L ( Kudo classification)

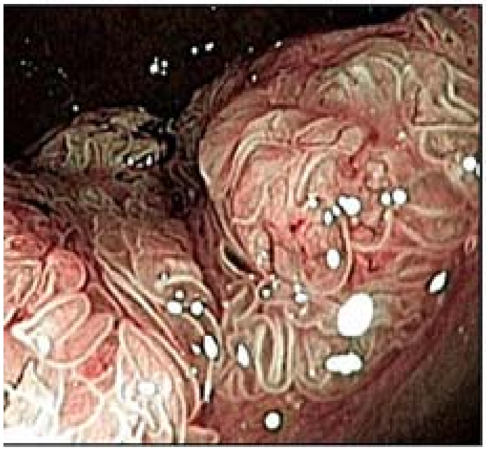

**Adenomatous polyp, type IV, NBI**

**FIGURE 18.** Colon: large adenomatous and villous polyp; narrow-band imaging coupled to magnification. Pit pattern classified as IV ( Kudo classification)

## Inflammatory Bowel Disease

The NBI technique is expected to contribute to the classification and re-evaluation of colitis through a fine description of the most early abnormalities at the surface and in the vascular network[21]. There is an increased risk of cancer in long standing ulcerative colitis and Crohn's disease and surveillance in endoscopy is recommended, unfortunately it has a poor efficacy in the early diagnosis of flat neoplastic areas in the inflammed mucosa because early alterations occur in the depth of the mucosa. Magnification with NBI should improve the exploration of suspect zones through the analysis of abnormal vascular patterns. As yet the studies are still anecdotal in one patient having dysplastic leskions in chronic ulcerative colitis and examined in NBI[23], the fine architecture of dysplastic areas differed from that of the non dysplastic mucosa and a high vascular density was observed in areas with a pit pattern type IIIL or IV.

Correlation between the alterations of the pit pattern and vascular pattern in inflammatory and dysplastic lesions was confirmed with histology.

## CONCLUSION

The NBI technique confers a further strength to the clinical utility of endoscopy in magnification in the esophagus, stomach, duodenum and large bowel. NBI, coupled to magnification offers an easy approach to the characterization of epithelial types (intestinal metaplasia) and the morphological analysis of epithelial ridges and grooves (pit pattern) at the surface of the mucosa. Its most important contribution is the clear vision of the vascular network of the mucosa. This applies to the evaluation of neo-angiogenesis in superficial digestive neoplastic lesions; classifications of the vascular abnormalities, from slight to severe, are being developed,with an attempt to T staging of those lesions. A benefit to our knowledge of the pathophysiology of inflammatory conditions of the digestive mucosa is also expected.
